# Supplementary material for: Urine Molecular Biomarkers for Detection and Follow-Up of Small Renal Masses
Source: Int J Mol Sci. 2022 Dec 17;23(24):16110. doi: 10.3390/ijms232416110 (PMC9785854; doi:10.3390/ijms232416110)
Supplement: Supplementary file 1 [file ijms-23-16110-s001.zip › ijms-2068048-supplementary.pdf]

Table S1. Associations of SRM growth rate with demographic and clinical demographic parameters.

Clinical demographic variable	Non-progressive SRMs (N=24)	Progressive SRMs (N=11)	p-value
Follow-up time (months)	20.5 (±10.1)	23.8 (±8.9)	0.334
Gender (Male vs. Female)	37.5% vs. 62.5%	54.5% vs. 45.5%	0.467
Age at diagnosis, years (Mean; ±SD)	77.0 (±7.5)	75.9 (±9.1)	0.612
Body mass index (Mean; ±SD)	29.2 (±5.9)	26.6 (±9.4)	0.687
Obesity (Yes vs. No)	45.8% vs. 54.2%	45.5% vs. 54.5%	1.00
PAH (Yes vs. No)	87.5% vs. 12.5%	100% vs. 0%	0.536
PAH duration, years (Mean; ±SD)	13.8 (±12.0)	15.5 (±11.2)	0.611
Diabetus melitus (Yes vs. No)	29.2% vs. 70.6%	27.3% vs. 72.7%	1.00
ECOG (Mean; ±SD)	1.2 (±1.0)	0.8 (±1.0)	0.334
Metabolic syndrome (Yes vs. No)	56.5% vs. 43.5%	30.0% vs. 70.0%	0.259
Previous cancer (Yes vs. No)	25.0% vs. 75.0%	9.1% vs. 90.9%	0.392
Charlston Comorbidity Index (Mean; ±SD)	4.8 (±1.8)	4.1 (±1.3)	0.334
Glomerular filtration rate, mL/min	61.1 (±20.1)	80.2 (±33.3)	0.140
Creatinine, µmol/L (Mean; ±SD)	97.4 (±59.8)	81.1 (±31.2)	0.524
Tumor histology (ccRCC vs. other subtypes)	58.3% vs. 41.7%	100% vs. 0%	**0.015**
ISUP grade (2 vs. 1)	75.0% vs. 25.0%	72.7% vs. 27.3%	1.00
Tumor volume, mm^3 (Mean; ±SD)			
First scan	6441.5 (±5803.3)	8052.4 (±6712.1)	0.370
Last scan	7013.8 (±7445.5)	21617.1 (±11068.1)	**<0.001**
Tumor maximal diameter, mm (Mean; ±SD)			
First scan	22.7 (±7.3)	24.6 (±7.4)	0.563
Last scan	23.1 (±8.2)	37.6 (±9.2)	**<0.001**
Change in tumor size during AS (Mean; ±SD)			
Volume	572.3 (±5016.2)	13564.7 (±6602.3)	**<0.001**
Diameter	0.4 (±5.1)	13.0 (±8.4)	**<0.001**

SRM – small renal mass; PAH – Pulmonary Arterial Hypertension; ECOG - Eastern Cooperative Oncology Group performance status; ccRCC – clear cell renal cell carcinoma; ISUP – International Society of Urological Pathology grade; SD – standard deviation.

Table S2. The diagnostic test performance characteristics (ROC analysis) of the analyzed methylation biomarkers in urine samples.

No.	Biomarker	AUC	95% of CI	P-value	Sensitivity, %	Specificity, %
**Single biomarker**						
4	*TFAP2B*	0.668	0.581 - 0.748	**0.001**	79.5	53.3
5	*TAC1*	0.676	0.589 - 0.755	**<0.001**	74.4	62.0
**3**	***PCDH8***	**0.690**	**0.604 - 0.768**	**<0.001**	**48.7**	**88.0**
1	*ZNF677*	0.582	0.492 - 0.667	**0.018**	20.5	96.7
6	*FLRT2*	0.559	0.470 - 0.646	0.128	23.1	90.2
2	*FBN2*	0.540	0.450 - 0.627	0.223	15.4	92.4
**Combination of two biomarkers**						
7	*ZNF677& FBN2*	0.587	0.498 - 0.672	**0.034**	28.2	90.2
8	*ZNF677 & PCDH8*	0.688	0.601 - 0.766	**<0.001**	51.3	84.8
9	*ZNF677 & TFAP2B*	0.684	0.597 - 0.762	**<0.001**	79.5	51.1
10	*ZNF677 & TAC1*	0.694	0.607 - 0.771	**<0.001**	74.4	62.0
11	*ZNF677 & FLRT2*	0.603	0.514 - 0.687	**0.018**	33.3	85.9
12	*FBN2 & PCDH8*	0.675	0.587 - 0.754	**<0.001**	53.9	83.7
13	*FBN2 & TFAP2B*	0.668	0.581 - 0.748	**0.001**	79.5	52.2
14	*FBN2 & TAC1*	0.666	0.578 - 0.746	**<0.001**	74.4	60.9
15	*FBN2 & FLRT2*	0.578	0.489 - 0.664	0.070	30.8	84.8
16	*PCDH8 & TFAP2B*	0.684	0.597 - 0.762	**<0.001**	79.5	52.2
**17**	***PCDH8 & TAC1***	**0.726**	**0.641 - 0.800**	**<0.001**	**84.6**	**57.6**
18	*PCDH8 & FLRT2*	0.691	0.605 - 0.769	**<0.001**	56.4	81.5
19	*TFAP2B & TAC1*	0.688	0.601 - 0.766	**<0.001**	89.7	40.2
20	*TFAP2B & FLRT2*	0.665	0.577 - 0.745	**0.001**	82.1	47.8
21	*TAC1 & FLRT2*	0.695	0.608 - 0.772	**<0.001**	82.1	55.4
**Combination of three biomarkers**						
22	*ZNF677, FBN2 & PCDH8*	0.675	0.588 - 0.754	**<0.001**	56.4	80.4
23	*ZNF677, FBN2 & TFAP2B*	0.683	0.596 - 0.761	**<0.001**	79.5	51.1
24	*ZNF677, FBN2 & TAC1*	0.684	0.597 - 0.762	**<0.001**	76.9	57.6
25	*ZNF677, FBN2 & FLRT2*	0.613	0.524 - 0.696	**0.015**	38.5	81.5
26	*ZNF677, PCDH8 & TFAP2B*	0.689	0.602 - 0.767	**<0.001**	69.2	60.9
27	*ZNF677, PCDH8 & TAC1*	0.721	0.636 - 0.796	**<0.001**	82.1	59.8
28	*ZNF677, PCDH8 & FLRT2*	0.694	0.608 - 0.772	**<0.001**	59.0	77.2
29	*ZNF677, TFAP2B & TAC1*	0.708	0.622 - 0.784	**<0.001**	76.9	55.4
30	*ZNF677, TFAP2B & FLRT2*	0.682	0.595 - 0.760	**<0.001**	82.1	45.7
31	*ZNF677, TAC1 & FLRT2*	0.708	0.622 - 0.784	**<0.001**	79.5	57.6
32	*FBN2, PCDH8 & TFAP2B*	0.683	0.596 - 0.761	**<0.001**	82.1	50.0
33	*FBN2, PCDH8 & TAC1*	0.713	0.627 - 0.788	**<0.001**	84.6	55.4
34	*FBN2, PCDH8 & FLRT2*	0.685	0.598 - 0.763	**<0.001**	59.0	80.4
35	*FBN2, TFAP2B & TAC1*	0.685	0.598 - 0.764	**<0.001**	79.5	51.1
36	*FBN2, TFAP2B & FLRT2*	0.672	0.585 - 0.752	**<0.001**	82.1	48.9
37	*FBN2, TAC1 & FLRT2*	0.691	0.604 - 0.769	**<0.001**	82.1	55.4
38	*PCDH8, TFAP2B & TAC1*	0.710	0.624 - 0.786	**<0.001**	79.5	56.5
39	*PCDH8, TFAP2B & FLRT2*	0.681	0.594 - 0.760	**<0.001**	92.3	38.0
**40**	***PCDH8, TAC1 & FLRT2***	**0.732**	**0.647 - 0.805**	**<0.001**	**89.7**	**54.4**
41	*TFAP2B, TAC1 & FLRT2*	0.697	0.610 - 0.774	**<0.001**	97.4	34.8

Table S2. Continued.

	**Combination of four biomarkers**					
42	*ZNF677, FBN2, PCDH8 & TFAP2B*	0.690	0.603 - 0.768	**<0.001**	71.8	59.8
43	*ZNF677, FBN2, PCDH8 & TAC1*	0.709	0.623 - 0.785	**<0.001**	87.2	52.2
44	*ZNF677, FBN2, PCDH8 & FLRT2*	0.691	0.604 - 0.769	**<0.001**	61.5	77.2
45	*ZNF677, FBN2, TFAP2B & TAC1*	0.702	0.616 - 0.779	**<0.001**	59.0	72.8
46	*ZNF677, FBN2, TFAP2B & FLRT2*	0.685	0.599 - 0.764	**<0.001**	82.1	46.7
47	*ZNF677, FBN2, TAC1 & FLRT2*	0.704	0.618 - 0.780	**<0.001**	82.1	54.4
48	*ZNF677, PCDH8, TFAP2B & TAC1*	0.717	0.632 - 0.792	**<0.001**	74.4	59.8
49	*ZNF677, PCDH8, TFAP2B & FLRT2*	0.685	0.599 - 0.764	**<0.001**	92.3	35.9
50	***ZNF677, PCDH8, TAC1 & FLRT2***	**0.736**	**0.652 - 0.809**	<0.001	**92.3**	**52.2**
51	*ZNF677, TFAP2B, TAC1 & FLRT2*	0.715	0.630 - 0.791	**<0.001**	71.8	60.9
52	*FBN2, PCDH8, TFAP2B & TAC1*	0.705	0.619 - 0.781	**<0.001**	87.2	46.7
53	*FBN2, PCDH8, TFAP2B & FLRT2*	0.685	0.599 - 0.764	**<0.001**	79.5	52.2
54	*FBN2, PCDH8, TAC1 & FLRT2*	0.728	0.643 - 0.802	**<0.001**	89.7	53.3
55	*FBN2, TFAP2B, TAC1 & FLRT2*	0.698	0.612 - 0.775	**<0.001**	84.6	48.9
56	*PCDH8, TFAP2B, TAC1 & FLRT2*	0.713	0.627 - 0.788	**<0.001**	100.0	33.7
	**Combination of five biomarkers**					
57	*ZNF677, FBN2, PCDH8, TFAP2B & TAC1*	0.711	0.625 - 0.786	**<0.001**	82.1	51.1
58	*ZNF677, FBN2, PCDH8, TFAP2B & FLRT2*	0.691	0.604 - 0.769	**<0.001**	79.5	51.1
59	*ZNF677, FBN2, PCDH8,TAC1 & FLRT2*	0.732	0.647 - 0.805	**<0.001**	92.3	51.1
60	*ZNF677, FBN2, TFAP2B,TAC1 & FLRT2*	0.714	0.629 - 0.790	**<0.001**	82.1	51.1
61	*ZNF677, PCDH8, TFAP2B, TAC1 & FLRT2*	0.723	0.638 - 0.797	**<0.001**	71.8	60.9
62	*FBN2, PCDH8, TFAP2B,TAC1 & FLRT2*	0.712	0.626 - 0.788	**<0.001**	89.7	44.6
	**Combination of all six biomarkers**					
63	*ZNF677, FBN2, PCDH8, TFAP2B, TAC1 & FLRT2*	0.722	0.637 - 0.796	**<0.001**	76.9	56.6

ROC – Receiver Operating Characteristic; AUC – area under the curve; CI – confidence intervals.

Table S3. Associations of biomarkers methylation level in patients diagnosed with SRMs urine samples with demographic and clinical characteristics.

**Clinical demographic variable**	***ZNF677***	***FBN2***	***PCDH8***	***TFAP2B***	***TAC1***	***FLRT2***
Gender (Male vs. Female)	Zad = -2.52; P = 0.092	Zad = -0.50; P = 0.769	**Zad = -2.64; P = 0.009**	**Zad = -2.04; P = 0.042**	Zad = -0.96; P = 0.347	Zad = -1.17; P = 0.392
Age at diagnosis (years)	Rs = 0.22; P = 0.185	Rs = 0.11; P = 0.488	Rs < 0.01; P = 0.991	Rs = 0.24; P = 0.144	Rs = 0.23; P = 0.156	Rs = 0.06; P = 0.717
Body mass index	Rs = -0.13; P = 0.429	Rs = 0.16; P = 0.352	Rs = 0.02; P = 0.887	Rs = -0.14; P = 0.404	Rs = 0.12; P = 0.520	Rs = -0.13; P = 0.449
Obesity (Yes vs. No)	Zad = 0.21; P = 0.897	Zad = 1.11; P = 0.496	Zad = 0.75; P = 0.478	Zad = -0.41; P = 0.696	Zad = 0.79; P = 0.443	Zad = 0.25; P = 0.874
PAH (Yes vs. No)	Zad = 0.83; P = 0.599	Zad = 0.76; P = 0.672	Zad = 0.11; P = 0.941	Zad = 1.27; P = 0.228	Zad = 1.59; P = 0.126	Zad = -0.71; P = 0.635
PAH duration (years)	Rs = 0.08; P = 0.640	Rs = 0.12; P = 0.539	Rs = 0.23; P = 0.184	Rs = 0.07; P = 0.705	Rs = 0.14; P = 0.408	Rs = -0.07; P = 0.672
Diabetus melitus (Yes vs. No)	Zad = -0.93; P = 0.548	Zad = -1.64; P = 0.316	Zad = 0.47; P = 0.656	Zad = -0.31; P = 0.770	Zad = -0.17; P = 0.866	Zad = -1.44; P = 0.301
ECOG	H = 3.27; P = 0.514	H = 1.77; P = 0.779	H = 5.38; P = 0.251	H = 1.61; P = 0.806	H = 2.45; P = 0.654	H = 4.55; P = 0.336
Metabolic syndrome (Yes vs. No)	Zad = -1.66; P = 0.287	Zad = -0.80; P = 0.613	Zad = -0.86; P = 0.405	Zad = -0.33; P = 0.757	Zad = 0.73; P = 0.482	Zad = -1.62; P = 0.245
Previous cancer (Yes vs. No)	Zad = -0.16; P = 0.929	Zad = -0.23; P = 0.900	Zad = 1.06; P = 0.322	Zad = 0.73; P = 0.484	Zad = 0.37; P = 0.734	Zad = -0.74; P = 0.603
Charlston Comorbidity Index	Rs = -0.02; P = 0.907	Rs = -0.06; P = 0.700	Rs = 0.05; P = 0.745	Rs = 0.09; P = 0.578	Rs = 0.10; P = 0.545	Rs = -0.28; P = 0.092
Glomerular filtration rate	Rs = -0.07; P = 0.695	Rs = 0.04; P = 0.836	Rs = 0.05; P = 0.757	Rs = -0.29; P = 0.091	Rs = -0.32; P = 0.062	Rs = 0.07; P = 0.695
Creatinine	**Rs = -0.33; P = 0.048**	Rs = 0.01; P = 0.970	**Rs = -0.36; P = 0.028**	Rs = -0.16; P = 0.332	Rs = 0.01; P = 0.943	Rs = -0.14; P = 0.406
Histology (ccRCC vs. other subtypes)	Zad = 0.12; P = 0.939	Zad = -0.25; P = 0.890	Zad = 0.34; P = 0.747	Zad = -0.56; P = 0.590	Zad = -1.13; P = 0.272	Zad = -0.95; P = 0.488
ISUP grade (2 vs. 1)	Zad = 0.46; P = 0.774	Zad = 0.15; P = 0.947	Zad = -0.23; P = 0.842	Zad = -0.72; P = 0.492	Zad = -0.53; P = 0.611	Zad = -0.39; P = 0.808
Tumor volume (mm^3)						
First scan, N=39	Rs = 0.14; P = 0.392	Rs = -0.28; P = 0.086	**Rs = 0.32; P = 0.051**	Rs = 0.15; P = 0.353	Rs = 0.15; P = 0.365	Rs = 0.11; P = 0.488
Second scan, N=37	Rs < -0.01; P = 0.990	Rs = 0.08; P = 0.659	Rs = 0.28; P = 0.100	Rs = -0.10; P = 0.581	Rs = 0.04; P = 0.803	Rs = -0.22; P = 0.195
Third scan, N=35	Rs = 0.06; P = 0.710	Rs = 0.18; P = 0.306	Rs = 0.13; P = 0.442	Rs = -0.20; P = 0.256	Rs = 0.09; P = 0.588	Rs = -0.09; P = 0.607
Fourth scan, N=26	Rs = 0.06; P = 0.791	Rs = 0.07; P = 0.754	Rs = -0.09; P = 0.668	Rs = -0.07; P = 0.760	Rs = -0.20; P = 0.347	Rs = -0.29; P = 0.167
Tumor maximal diameter (mm)						
First scan, N=39	Rs = 0.20; P = 0.222	Rs = -0.28; P = 0.088	**Rs = 0.35; P = 0.028**	Rs = 0.26; P = 0.107	Rs = -0.12; P = 0.475	Rs = 0.19; P = 0.255
Second scan, N=37	Rs = 0.03; P = 0.879	Rs = 0.13; P = 0.461	Rs = 0.28; P = 0.098	Rs = -0.10; P = 0.566	Rs = 0.04; P = 0.839	Rs = -0.21; P = 0.224
Third scan, N=35	Rs = 0.08; P = 0.666	Rs = 0.16; P = 0.368	Rs = 0.13; P = 0.458	Rs = -0.17; P = 0.319	Rs = 0.11; P = 0.534	Rs = -0.04; P = 0.814
Fourth scan, N=26	Rs = 0.04; P = 0.867	Rs = -0.09; P = 0.694	Rs = -0.05; P = 0.814	Rs = -0.09; P = 0.682	Rs = -0.18; P = 0.403	Rs = -0.27; P = 0.198
Change in tumor size during AS						
Volume	Rs = 0.03; P = 0.847	Rs = -0.14; P = 0.399	Rs = 0.01; P = 0.933	**Rs = -0.33; P = 0.048**	Rs = -0.19; P = 0.262	**Rs = -0.41; P = 0.012**
Diameter	Rs = -0.09; P = 0.593	Rs = -0.15; P = 0.384	Rs = -0.06; P = 0.722	**Rs = -0.47; P = 0.004**	Rs = -0.22; P = 0.201	**Rs = -0.50; P = 0.002**
Tumor progression (Yes vs. No)	Zad = -0.11; P = 0.958	Zad = -1.61; P = 0.334	Zad = -0.70; P = 0.517	**Zad = -2.38; P = 0.016**	Zad = -1.18; P = 0.252	Zad = -1.96; P = 0.174

Rs - Spearman correlation coefficient; Zad – Mann-Whitney's Z adjusted parameter; H – Kruskal-Wallis's H parameter.

Table S4. Demographic and clinical-pathological characteristics of the patients diagnosed with small renal masses (N=39).

Parameter	N (%)	Mean	Median	Min	Max	IQR
**SRM (pT1a) Patients**	39 (100.0%)	-	-	-	-	-
**Follow-up time**	-	20	22	0	41	12-27
**General information and medical history**						
**Gender**						
Male	17 (44.0%)	-	-	-	-	-
Female	22 (56.0%)	-	-	-	-	-
**Age at diagnosis**	-	76	78	59	93	72-81
**Smoker**						
No	33 (84.6%)	-	-	-	-	-
Current	2 (5.1%)	-	-	-	-	-
Former	4 (10.3%)	-	-	-	-	-
**Body mass index**	-	29.9	29.6	17.6	42.9	26.4-33.1
Normal						
High						
**PAH**						
No	3 (7.7%)	-	-	-	-	-
Yes	36 (92.3%)	-	-	-	-	-
Duration	-	13.8	10.0	0.0	45.0	5.5-20.0
**Diabetus melitus**						
No	28 (71.8%)	-	-	-	-	-
Yes	11 (28.2%)	-	-	-	-	-
**Other/previous cancers**						
No	33 (84.6%)	-	-	-	-	-
Yes	6 (15.4%)	-	-	-	-	-
**Charlson Co-morbidity Index**	-	4.4	4.0	2.0	9.0	3.5-5.0
**ECOG scale**						
0	13 (33.3%)	-	-	-	-	-
1	17 (43.6%)	-	-	-	-	-
2	5 (12.8%)	-	-	-	-	-
3	3 (7.7%)	-	-	-	-	-
4	1 (2.6%)	-	-	-	-	-
**Metabolic syndrome points**	-					
0	1 (2.6%)	-	-	-	-	-
1	8 (20.5%)	-	-	-	-	-
2	10 (25.6%)	-	-	-	-	-
3	13 (33.3%)	-	-	-	-	-
4	5 (12.8%)	-	-	-	-	-
5	2 (5.1%)	-	-	-	-	-

Table S4. Continued.

		**Clinical-pathological tumor parameters**				
**Tumor histology**						
ccRCC	28 (71.8%)	-	-	-	-	-
pRCC	7 (17.9%)	-	-	-	-	-
ccRCC/pRCC	1 (2.6%)	-	-	-	-	-
chRCC	3 (7.7%)	-	-	-	-	-
**Tumor growth**						
Stable	24 (61.5%)	-	-	-	-	-
Rapid	11 (28.2%)	-	-	-	-	-
Unknown	4 (10.3%)	-	-	-	-	-
**Tumor maximal diameter (mm)**						
First scan	-	23.3	22.6	10.0	37.0	17.8-28.0
Last scan	-	27.6	28.0	12.0	52.0	19.0-34.0
**Tumor volume (mm³)**						
First scan	-	7187.3	4810.7	533.0	20860.0	2300.0-10134.6
Last scan	-	11629.1	10081.1	517.7	44131.8	2607.6-16929.6
**ISUP grade (ccRCC and pRCC only)**						
G1	10 (27.8%)	-	-	-	-	-
G2	23 (63.9%)	-	-	-	-	-
Unknown	3 (8.3%)	-	-	-	-	-
		**Kidney functions**				
**Glomerular filtration rate (mL/min)**	-	71.7	67.0	21.7	149.0	53.3-80.5
**Creatinine (μmol/L)**	-					
Male	-	108.0	87.0	72.0	287.0	78.5-102.0
Female	-	78.6	67.0	52.0	243.0	57.0-79.5

IQR - interquartile range; SRM – small renal mass; PAH – Pulmonary Arterial Hypertension; ECOG - Eastern Cooperative Oncology Group performance status; ccRCC – clear cell renal cell carcinoma; pRCC – papillary RCC; chRCC – chromophobe RCC; cc/pRCC – mixed (clear cell and papillary) histology RCC; ISUP – International Society of Urological Pathology grade.

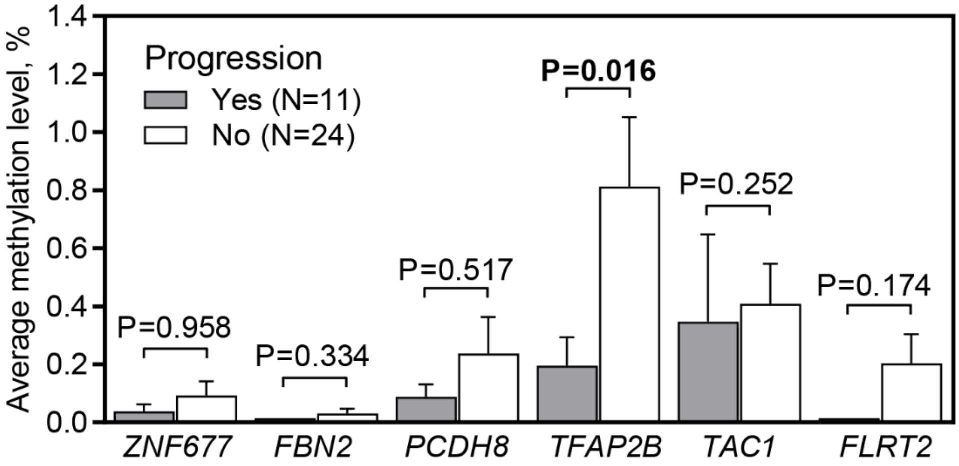

Figure S1. Methylation levels of investigated biomarkers according to progressiveness of SRMs. The association of methylated *ZNF677*, *FBN2*, *PCDH8*, *TFAP2B*, *TAC1*, and *FLRT2* levels at SRMs diagnosis with tumor progression. Significant P-values are in bold.

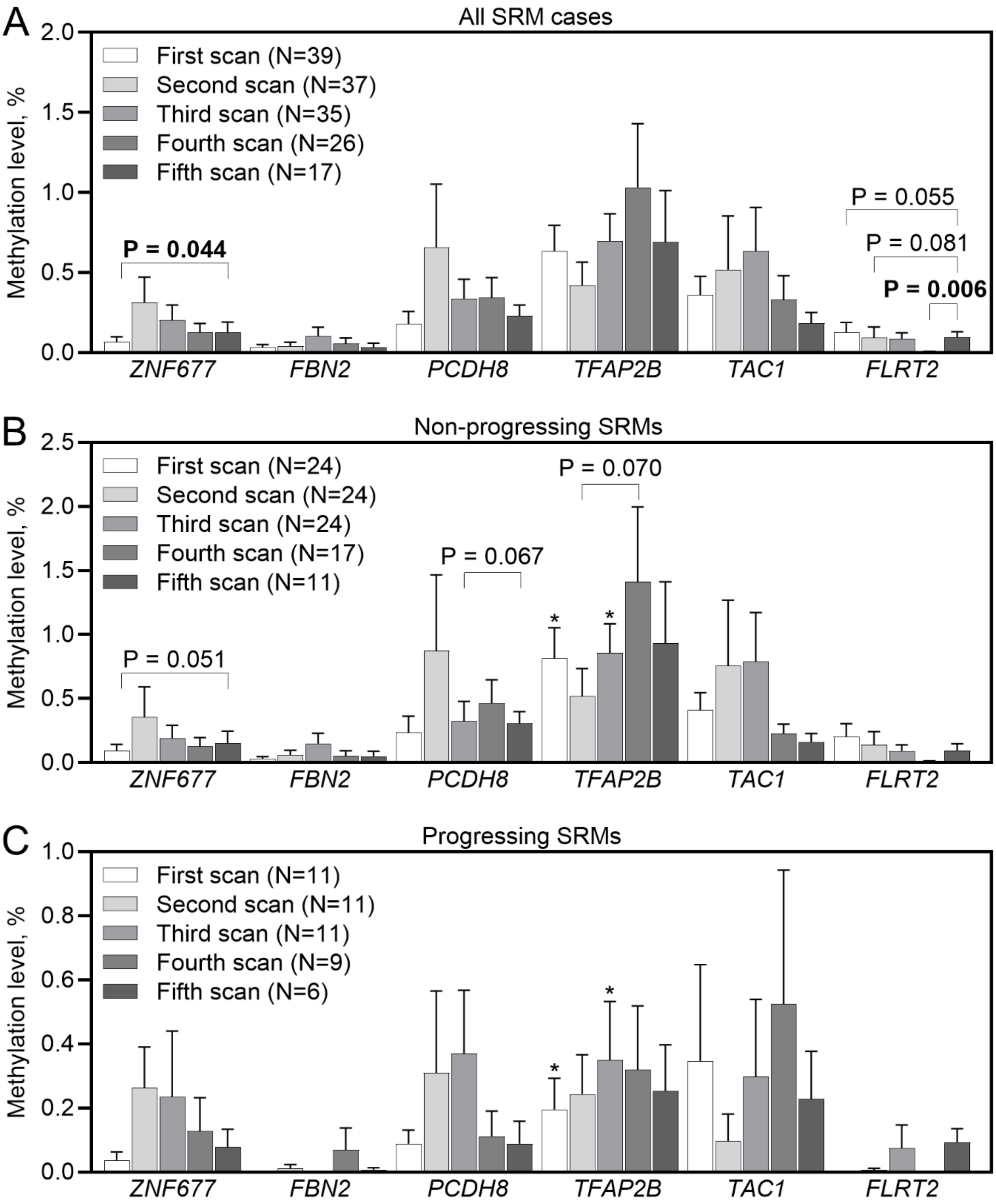

Figure S2. Changes in the methylation levels of the investigated biomarkers at the different time-points during the patient's follow-up. A – Methylated levels of *ZNF677*, *FBN2*, *PCDH8*, *TFAP2B*, *TAC1*, and *FLRT2* at different time points of active surveillance in all investigated SRM cases. B – Methylated levels of *ZNF677*, *FBN2*, *PCDH8*, *TFAP2B*, *TAC1*, and *FLRT2* at different time points of active surveillance in non-progressing SRM cases. C – Methylated levels of *ZNF677*, *FBN2*, *PCDH8*, *TFAP2B*, *TAC1*, and *FLRT2* at different time points of active surveillance in progressing SRM cases. * – depicts significant differences in methylation levels between non-progressing and progressing cases at a particular time point. Significant P values are in bold.

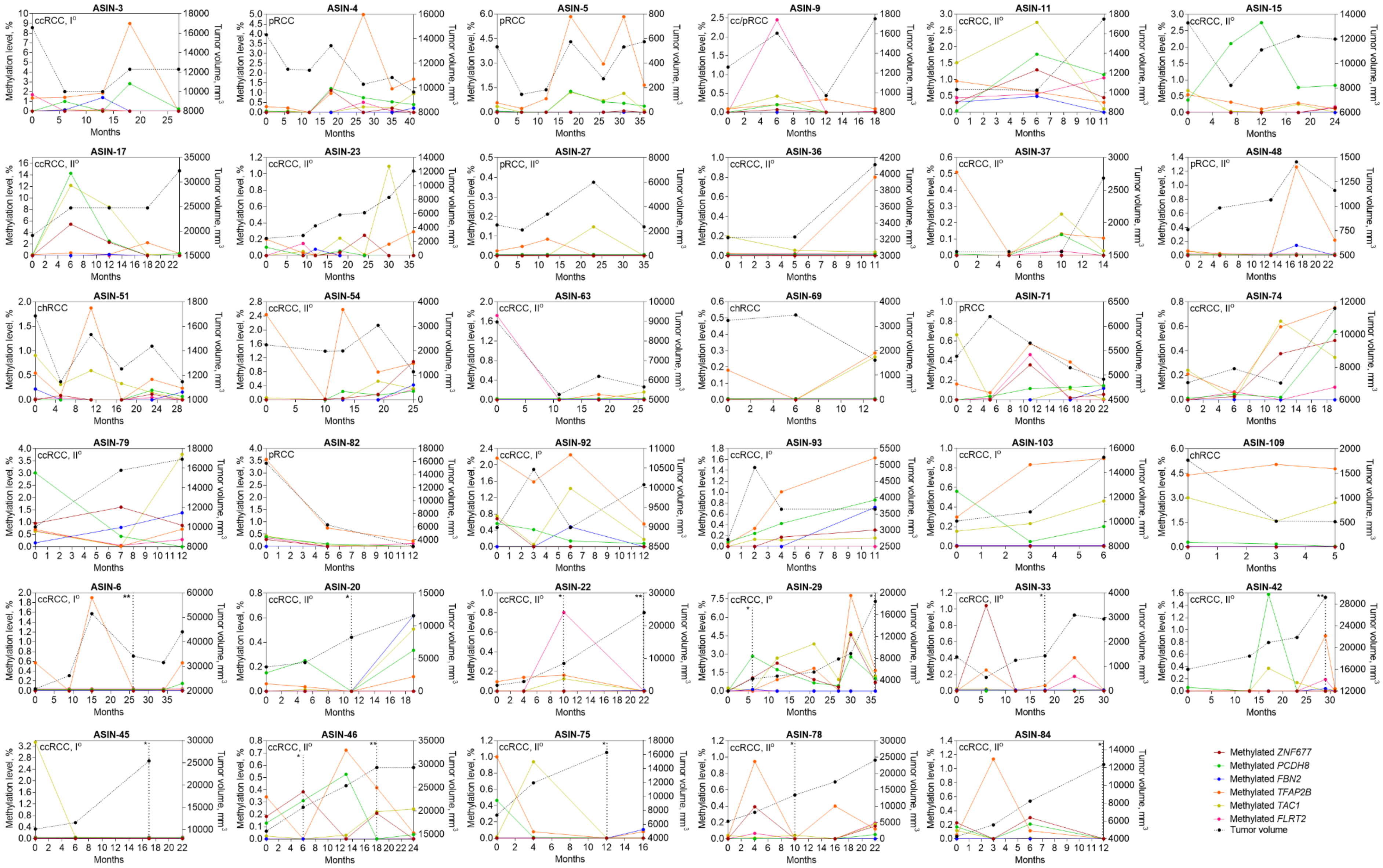

Figure S3. Associations between individual tumor growth dynamics and methylated levels of investigated genes. ccRCC – clear cell renal cell carcinoma; pRCC – papillary RCC; chRCC – chromophobe RCC; cc/Prcc – mixed (clear cell and papillary) histology RCC. ASIN depicts the patient's ID. A vertical dotted line depicts the tumor exceedance of the 4 cm limit in maximal diameter(*) or the doubling of tumor volume (**).
